# A New York Oculist

**Published:** 1899-02

**Authors:** 


A New York oculist is reported in a scientific journal as saying "that Americans are too reckless of their eyes in exposing them to the electric light rays, and if a reform is not had, a sightless race may be developed." "The proper thing to do," he adds, "is to abolish electric lamps altogether, and substitute fluorescent tubes, which cost no more and give a steady light." It is said that arrangements are being made to light an entire block in New York by fluorescent tubes.—*N. Y. Med. Times.*

